# Providing telenursing care for victims: a simulated study for introducing of possibility nursing interventions in disasters

**DOI:** 10.1186/s12911-022-01792-y

**Published:** 2022-03-02

**Authors:** Mahdiye Nejadshafiee, Mahmoud Nekoei-Moghadam, Kambiz Bahaadinbeigy, Hamidreza Khankeh, Hojjat Sheikhbardsiri

**Affiliations:** 1 Student Research Committee, Kerman University of Medical Sciences, Kerman, Iran; 2 Health in Disasters and Emergencies Research Center, Institute for Futures Studies in Health, Kerman University of Medical Sciences, Kerman, Iran; 3 Medical Informatics Research Center, Institute for Futures Studies in Health, Kerman University of Medical Sciences, Kerman, Iran; 4 Department of Educational and Rehabilitation Psychology, Leipzig University, Leipzig, Germany

**Keywords:** Telenursing, Telemedicine, Simulation, Victim, Nursing, Intervention, Disaster

## Abstract

**Introduction:**

Uncertainty occurrence of disasters requires special attention and a shortage of health care specialists is a challenge for health care systems; therefore, the use of telenursing care during a disaster is an appropriate way to provide care. This study aimed to investigate telenursing operational possibilities in disasters.

**Methods:**

A cross-sectional study was performed by implementing a functional exercise (Drill) for the possibility of nursing interventions in the response phase to disasters at Kerman University of Medical Sciences in 2021. Two evaluators examined and scored the possibility of providing telenursing care using a researcher-made checklist and we surveyed Inter-rater agreement between two evaluators by Cohen's kappa coefficient. Data were analyzed using descriptive tests and SPSS 20 software.

**Results:**

Findings showed that implementation of telenursing care would be helpful in future disasters. The scores received from assessment of the evaluation checklist for this simulated exercise program by the first evaluator was 83.25 and for the second evaluator was 72.00. The results of the study showed that the mean score of the possibility of telenursing in disasters was at a high level 77.50. Thus, the quality of the telenursing care in simulated conditions was satisfactory.

**Conclusion:**

Today, disaster management is almost impossible without using new technologies. This study found that due to the lack of specialized nursing staff in the deprived areas affected by disasters, the most important way to provide health care for a large group of the population is to develop effective health services so that everyone can use these services equally and fairly.

## Introduction

Disasters pose severe threats to the life, development and evolution of human society [1]. Unexpected incidents and disasters and the resulting damage are increasing due to environmental changes, economic, social and political factors [2]. Among all the consequences of these disasters, health is the most important priority of any society [3]. Therefore, it is essential to pay attention to the prevention, forecasting and provision of necessary supplies and equipment to provide an effective and appropriate response to reduce mortality, injury, disability and the burden caused by these disasters [4].

**Fig. 1:**
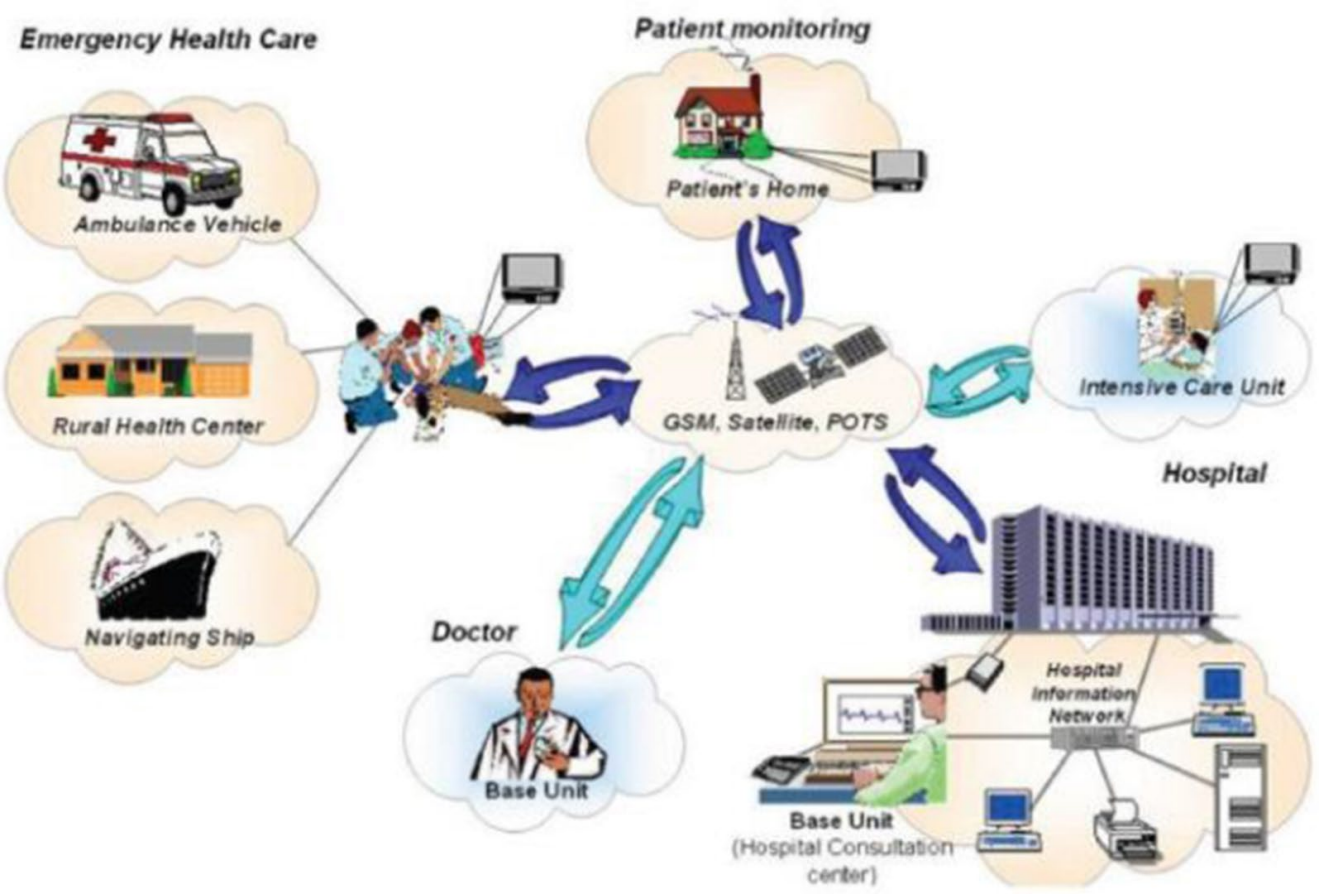
Telemedicine system architecture

Telemedicine, which is often used interchangeably with telehealth, is defined as the delivery of health care services over the internet for "the diagnosis, treatment, prevention of disease and injuries, research and evaluation, and education of health care providers" to improve health [5]. Figure 1, describes the overall system architecture [6]. The National Air and Space Administration (NASA) used wireless telemedicine for the first time in the 1985 Mexico City earthquake, which destroyed all terrestrial communication infrastructures. Voice transmission via advanced communications satellite (ATS-3) was the only option available to international rescue organizations. In addition, a space bridge was constructed in Armenia during the earthquake and in those days, the virtual bridge enabled global satellite communications between various countries, including Russia and the United States [8]. Telemedicine, with its distinct features, can respond quickly to critical needs in the event of a disaster [11]. For example, in the event of an infectious disease outbreak, remote tracking of care can be quickly and easily implemented using information technology [12]. Evidence suggests that there has been a significant increase in the use of multiple cases of successful telemedicine in multinational disasters [5]. With the COVID-19 pandemic of 2020, however, the application of telemedicine and its ability to provide safe, rapid, and high-quality care is apparent [14].

Nurses, who are the first care providers, coordinators, trainers and information providers and play a dynamic role in disasters [7]. Telenursing can accelerate the development of nursing and health because it seems that information and communication technology can provide an appropriate solution for the training of professionals, research, management and supplies, monitoring and quality assurance in health care [8, 9]. Telenursing has the potential to support effective disaster response, it includes medical examinations of the injured people, remote observations of graphs, tests and specialized consultations and in the recovery phase, it contains psychiatric counselling and rehabilitation for the disabled people [10]. Schools of nursing can increase inter-professional disaster training opportunities by expanding their use of educational technology, such as telenursing and virtual simulations in collaboration with community disaster response agencies [11].

## Literature review

Rizk and Siam investigated the effect of telenursing education on nurses' compliance with standard precautions during the COVID-19 pandemic. They concluded that the application of the telenursing education program had a positive impact in improving the compliance with the standard precautions scale (CSPs) of the studied nurses, as telenursing appears to be a new opportunity in the COVID-19 Pandemic disaster to reduce the risk of infection [12]. A study was conducted in South Korea, to develop the first telenursing service for covid-19 patients and declared that policies for appropriate and prompt healthcare interventions in disasters through telenursing should be designed to respond to the rapidly changing healthcare environment [13]. Mahdiye Nejadshafiee et al. in a study with title telenursing in incidents and disasters: a systematic review of the literature and their presented application of telemedicine in the tree component including clinical teams, disaster and communication types, and key outcomes of the telehealth programs used in disasters and incidents. Furthermore, the findings indicated that providing health care during a disaster is critical and that technology is essential for such care. Because of the scarcity of specialized nurses in disaster areas, including such a group in the telehealth program will open up a new avenue for care. As a result, telenursing provides a means of improving healthcare response [9]. Salehinejad et al. in a systematic review study looked into the use of telemedicine and e-health systems in the four stages of disaster management: mitigation and prevention, preparedness, response, and recovery. According to their findings, the majority of studies used telemedicine in the post-disaster or recovery phase. Shortly after the response phase, local governments attempt to rehabilitate residents and return them to their normal lives [14].

Injured people usually require a wide range of care during disasters. On the other hand, the weak nursing knowledge, attitude and skill in disasters, as well as according to the shortage of specialized nurses in disaster areas, so the use of telenursing care can be considered as an excellent way to provide care. Limited research has been carried out on telenursing care in disasters. Hence, the frequency of disasters has increased in Iran and no study has investigated the outcomes of using telenursing care for injured people in disasters, so this study aimed to assess the possibility of providing telenursing care in disasters through implementing a functional exercise.

## Method

The Ethical Committee of Kerman University of Medical Sciences approved this study before collecting the data, a cross-sectional study was used in 2021. We in this study designed an exercise drill in simulated conditions to investigate telenursing operational possibility in disaster.

### Study participants and sampling

Given that, the selection of members participating in the exercise should be selected based on the type, scope and levels of exercise [15], therefore exercise participants were selected based on purposive sampling methods. We invited specialists in the field of nursing, health in disasters and emergencies and health information technology (HIT) to perform and evaluate this drill. The number of staff that participated in the drill exercise included exercise executors (research team, n = 3), Actors team (hospital emergency department nurses, n = 6), Player (maquettes, n = 6), Controllers (two nursing experts with experience in disasters and two HIT experts, n = 4) and evaluators (two experts of health in disasters and emergencies, n = 2).

### Inclusion and exclusion criteria

Inclusion criteria were willingness to participate in exercise and have at least a bachelor's degree in nursing and health information technology. The exclusion criterion was the unwillingness to cooperate and continue the research.

### Exercise scenario and content

The scenario involved a simulated 6-magnitude earthquake occurring in Kerman city. This earthquake according to the texture, type of building and the population living in the area has caused human and financial losses as well as health consequences and seeks treatment. News of initial observations shows that more than 200 people were injured.

Based on the facilities available at the clinical skills center in the school of nursing and midwifery in Kerman, we assessed six maquettes as hypothetical victims to assess the feasibility of telenursing interventions in disasters. This study was part of the research dissertation of the research team that was conducted to obtain a doctorate degree in health in disasters and emergencies at the Kerman University of Medical Sciences. In this thesis, the research team extracted telenursing interventions through reviewing the literature, systematic review [9]and interviewing with nursing experts with experience in disaster in Iran, qualitative study [16]. Therefore, we wanted to evaluate the feasibility of performing these nursing interventions in the simulated environment.

### Perform of drill

After necessary coordination with nurses, professors, experts of the information technology (IT) department and clinical skills center, an operations-based exercise was done at the clinical skills learning center of Kerman University of Medical Sciences to evaluate the possibility of these simulated services with the technological infrastructure available in Kerman University of Medical Sciences. This scenario included the earthquake scale, the number of victims, the affected area, types of injuries and the capacity of the local hospital. The nursing care services were provided for six hypothetical casualties during a disaster in Kerman. Considering that after the occurrence of natural disasters such as earthquakes, most of the injured suffer from various traumas, the nurses present in the exercise site, who were working in teaching hospitals of Kerman, sent the information related to hypothetical injured to experienced nurses by the equipment available at the exercise site (Internet) and provided the target care after receiving their responses.

**Fig. 2:**
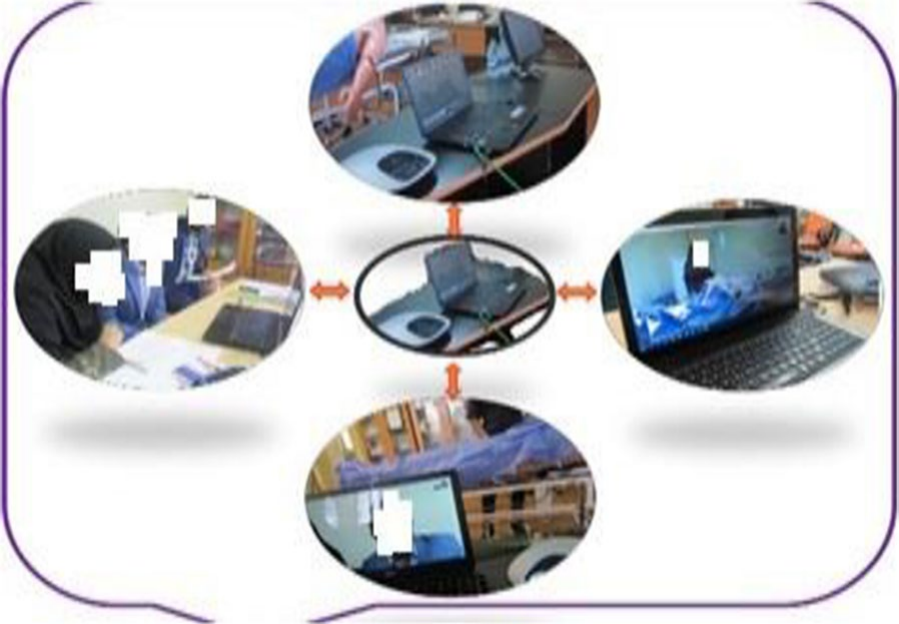
Exercise architecture

**Fig. 3:**
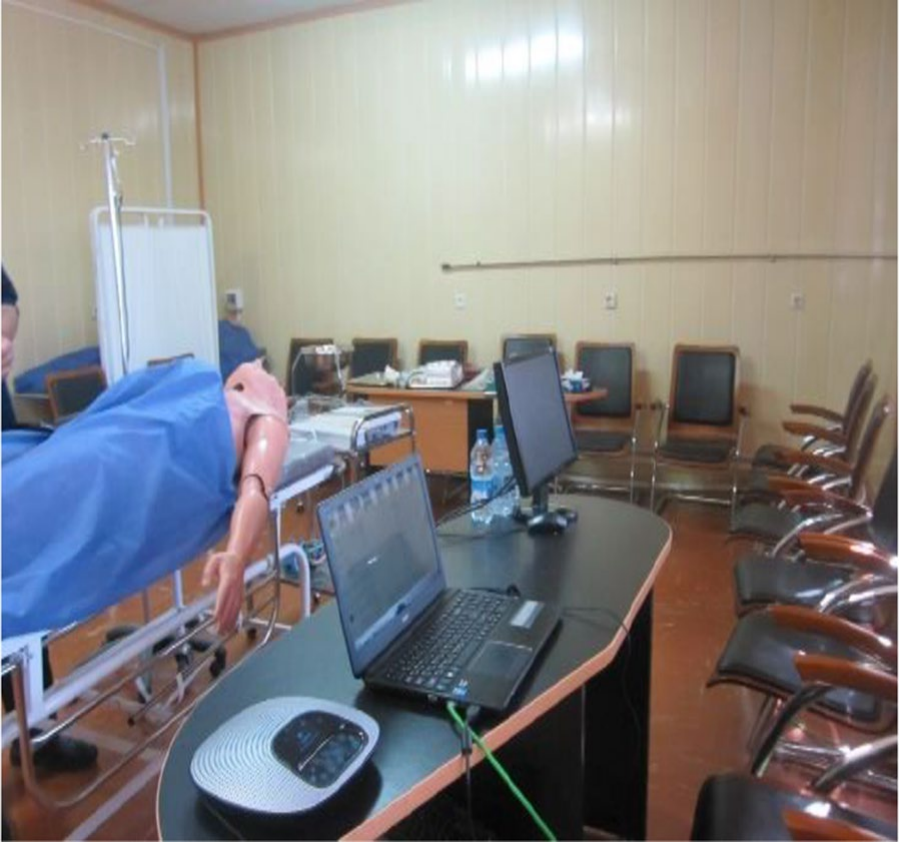
The systems utilized in the disaster response exercise

**Fig. 4:**
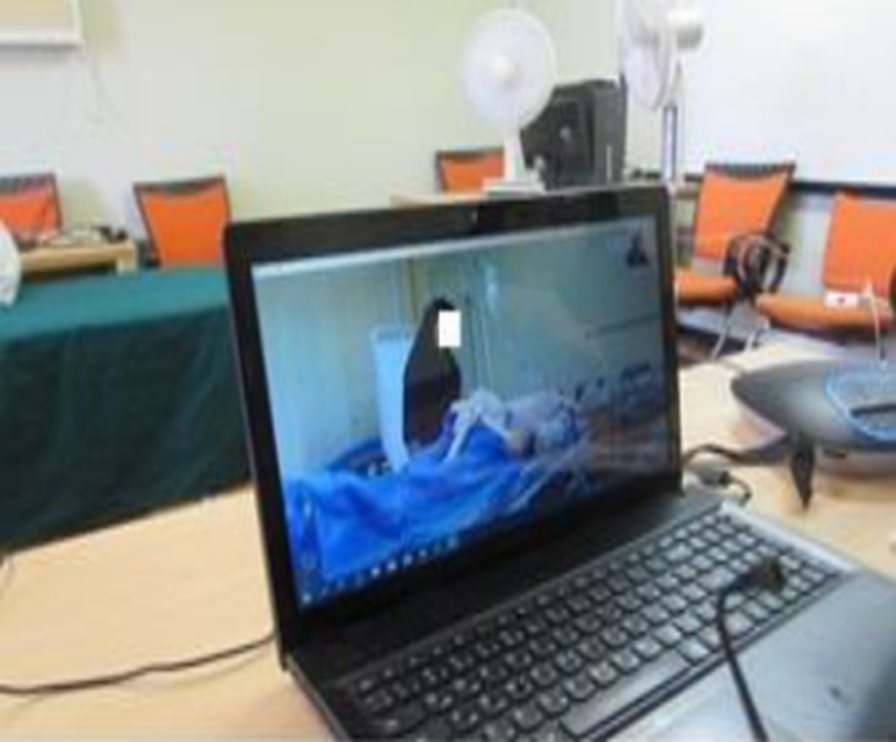
Part of the site disaster response field exercise included casualties from earthquake

**Fig. 5:**
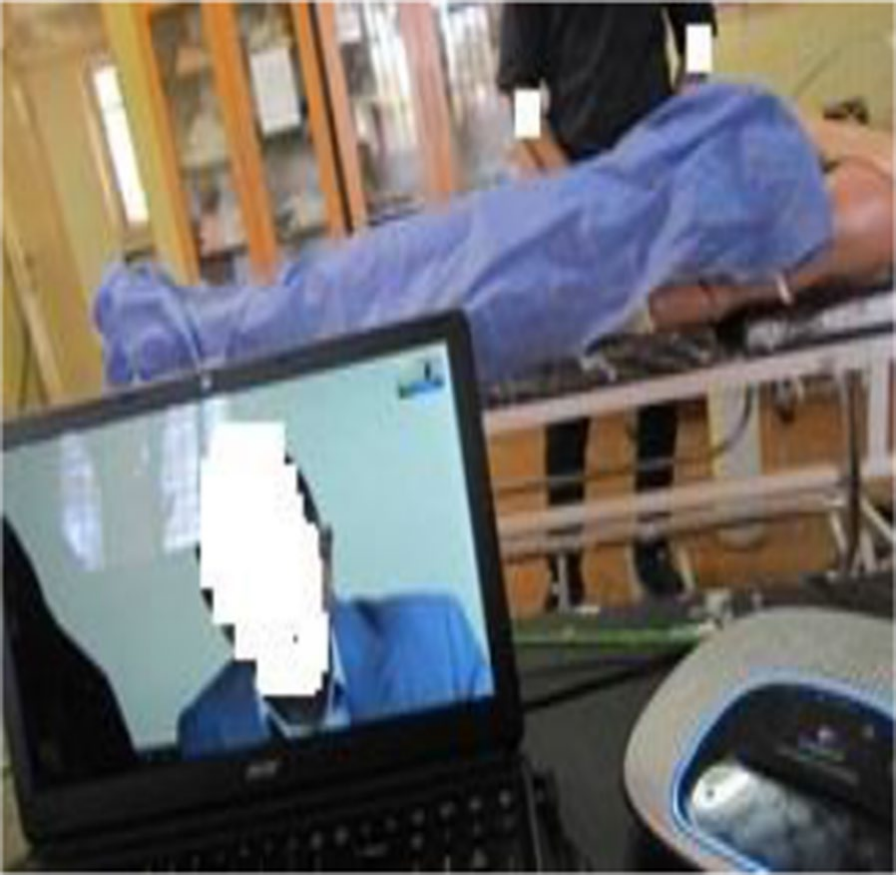
Casualty guidance by experienced nurses

**Fig. 6:**
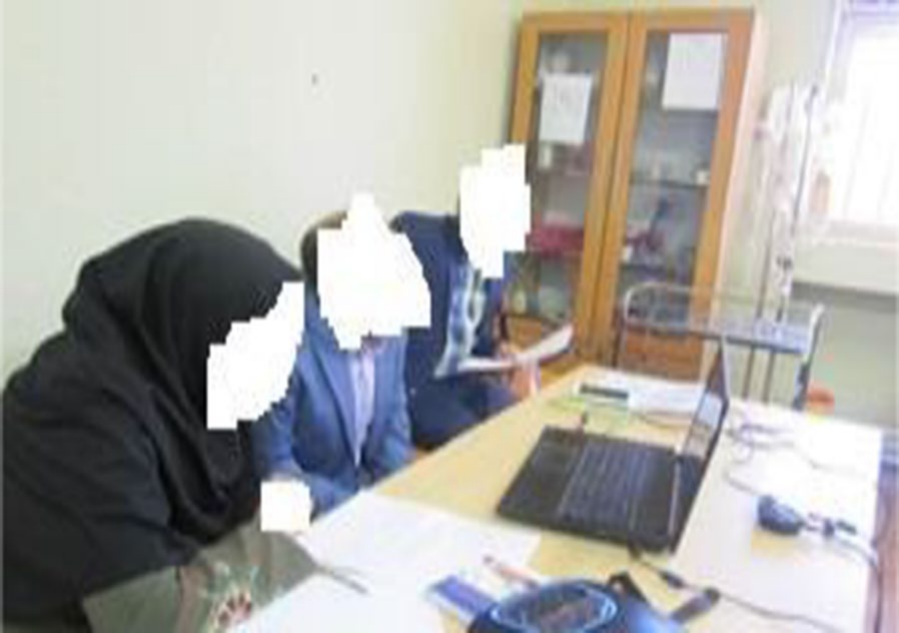
Casualty evaluation by Exercise observant

In this study, the researcher examined the possibility of providing care for trauma victims in the form of a scenario and asked observers to evaluate its possibility (Figs. 2,3,4,5 and 6). To respond to the earthquake, six nurses of Afzalipour hospital and two nurses experienced in disasters, who were professors of Razi School of Nursing and Midwifery in Kerman, were trained regarding the use of telecommunication equipment (Skype software), disaster scenario, general description and process of the program twenty minutes before the start of the exercise. Most victims suffer from various traumas after natural disasters such as earthquakes. In this scenario, in case the nurses available in the clinical skills learning center of Kerman University of Medical Sciences were uncertain or did not have sufficient knowledge of providing care, they using the available technology consulted qualified nurses, who were present in a place far from the injured. Then they immediately operationalized the received response and provided the correct care. Case scenarios were right arm fracture, hypovolemic shock, start triage, spinal cord trauma, crush injury and infant burns.

### Data collection

For data collection, the research team initially developed a checklist for assessing the possibility of nursing interventions in disasters after reviewing related textbooks, interviewing experts, and reading the Iranian emergency operation plan (EOP). We performed a preliminary validation for the preparation checklist and performed the stages of validation including face and content validity and reliability. Participants in this stage for completing of steps checklist validity (face and content validity) included nurses who had experience responding to disasters and we selected ten nurses for each stage. Six tabletop drills at educational hospitals were chosen for this study to test instrument reliability. Two evaluators evaluated each drill independently. After the evaluators completed their evaluations of all drills, the collected data was entered into SPSS 20 and the reliability of the instrument was examined using internal consistency and Intraclass Correlation Coefficient (ICC). The survey Inter-rater agreement between two evaluators was determined by Cohen's kappa coefficient.

## Results

The hypothetical victims characterize and predicted equipment are shown in Table 1. The results validation of the study checklist to begin, seven items were corrected for spelling and no items were removed during the qualitative face validity stage. Following the calculation of the impact score, 33 items with impact scores ≥ 1.5 were considered favorable and kept for further analysis in the content validity stage.

**Table 1:** Hypothetical victims characterize and predicted equipment

Introduce of case	Required equipment
A Conscious 30-year-old man with right arm fracture and deformity observed in left wrist, open airway and normal breathing, No symptoms of external bleeding, Normal vital signs Vital Signs: BP:110/60, HR: 68, RR: 16	Types of bandages and casts, sphygmomanometer, peripheral venous catheter, serum
A 24-year-old woman with lethargy, severe bleeding from the arm, fracture ribs and visible wounds on the chest, GCS 11, asymmetric pupil, laceration in forehead, have respiratory distress, weak and fast pulse Vital Signs: HR: 122, BP: 108/70, RR: 35	Oxygen, peripheral venous catheter, serum Zinc oxide adhesive plaster, Resuscitation equipment’s, Pulse oximetry, IV Stand, Tourniquet, Disposable Infusion set, Oxygen Mask, Oxygen Capsule
A 22-year-old man is conscious and have trauma to left femoral and open compound fracture. Respiratory rate above 30, capillary return less than 2 s and It responds to pain and the airway is open Vital Signs: HR: 130, BP: 70/30, RR: 35	Triage card, peripheral venous catheter, serum, Disposable Infusion set, Splint and flashlight
A 45-year-old man with a spinal cord injury was unable to move his arms and legs , Neck pain, low blood pressure and is bradycardia Vital Signs: HR: 66, RR:14, BP: 96/70	Long-backboard, cervical collar, serum, Disposable Infusion set, peripheral venous catheter and flashlight
A 50-year-old man emerged from under the rubble with extensive crushing on both lower extremities, ecchymosis and severe edema in the affected, pain; the pulse is not touched in the affected areas, disease background with a vague kidney, alert and included normal vital signs Vital Signs: HR: 150, BP: 145/75, RR: 16	Mannitol serum, monitoring devise, drugs, Disposable Infusion set, IV Stand, chest lead, pansement equipment’s and syringe
A 5-year-old child with weighs 20 kg, burn on one hand and the front of the chest, restless, large blisters on the limbs Vital Signs: HR: 150, BP: 95/65, RR: 25	Oxygen, Oxygen Mask, Oxygen Capsule, Disposable Infusion set, peripheral venous catheter, Normal saline or ringer serum, Burn pansement equipment’s

*BP* blood pressure, *HR* heart rate, *RR* respiratory rate, *GCS* Glasgow Coma Scale

To determine the qualitative content validity, most of the items were transcribed by adding, reducing or replacing some words with more familiar and understandable terms, which led to the clarification of ambiguous items. Items with the same implications were removed and some of them were merged because of overlapping. At these stages, eight items were merged into other items, and 25 items were kept for further analysis. Based on the opinions of 10 experts, the CVR was considered significant during the quantitative content validity stage (> 0.59). Thus, three items were removed. At the end of this stage, the number of items in the questionnaire was reduced to 22 ones. According to Polit, five items with Kappa coefficients less than 0.74 were omitted [17], bringing the total number of items to 17. Then, based on the mean CVI scores of all items (S-CVI/Average), the mean CVI of the whole tool was calculated, with 0.97 being the acceptable standard [18]. In the reliability section ICC = 0.87 test indicated that the evaluation checklist completed by two evaluators was also reliable. Based on Cohen's Kappa statistic test results showed the Inter-rater agreement between two evaluators was 0.87 and it was good.

The checklist contained 17 items and assessed the nurses' performance in taking care of the injured, and the evaluators were asked to rate each item on a scale of 1 (strongly disagree) to 5 (strongly agree). The minimum score was 17 and the maximum was 85. The few possibilities are defined as 17–39.6 points, the moderate possibility is 39.7–62.2 points, and the high possibility is 62.3 to 85 points.

### Evaluation results

Finding showed that implementation of telenursing care would be helpful in future disasters. The scores obtained from the review of the evaluation checklist for this simulated exercise program by the first evaluator was (83.00) and for second evaluator was (72.00). The results of the study showed that the mean score of possibility of telenursing in disasters was at a high level (77.50). Thus, the quality of the telenursing care in simulated condition was satisfactory, Showed in Table 2.

**Table 2:** Evaluating the practice of feasibility of nursing interventions in disasters based on the answers of two evaluators

Items	First evaluator	Second evaluator
	Completely disagree = 1, Disagree = 2, agree to some extent = 3, agree = 4 and strongly agree = 5	
(1) In the simulated scenario, audio signal was established at the determined time	5	4
(2) In the simulated scenario, video signal was established at the determined time	5	4
(3) Mutual communication and interaction were clear and transparent	5	3
(4) Nurses used the available communication and information tools easily	5	3
(5) In this scenario, the nurses were well acquainted with their duties	5	4
(6) According to the training given to nurses on how to use communication and information tools before the scenario, they were able to use Skype software	5	3
(7) Nurses' guidance in caring for the hypothetical injured was useful and helpful and they were able to provide the care for the patients. Following the care instructions, the nurse immobilized the fractured arm of the patient with a fiberglass cast	5	5
(8) Expert nurses sent all instructions on how to care for a shocked patient and the relevant nurse operationalized them well	5	4
(9) The expert nurse provided adequate explanations about the triage of the patient No.3 and emphasized the need for emergency care and treatment of the injured	5	5
(10) The nurse caring for a patient with spinal cord trauma could establish audio–video contact with expert nurses and received the necessary instructions on how to limit the movement of neck with cervical collar properly	5	4
(11) The hypothetical patient had extensive crush injury to the lower limbs. The nurse did not have sufficient knowledge and experience in caring for the injured, so she could provide care to the injured with remote guidance and prevented further irreparable complications	5	5
(12) The nurse caring for a child with burns of the anterior chest and one arm implemented the initial measures well under the supervision of expert nurses	5	4
(13) Nurses were able to provide the care to patients	4	4
(14) Nurses used the instructions of expert nurses	5	5
(15) In this scenario, telenursing led to time conservation	4	5
(16) According to the raters, the use of telenursing was a valuable source of information for inexperienced nurses	5	5
(17) Patient care was well managed	5	5
Total scores = 77.50	83 = High possibility	72 = High possibility

### Exercise hot wash stage

After the exercise, a joint meeting was held again with the presence of the research team, technology experts and the exercise team at the exercise site and the problems, weaknesses and strengths of the exercise were discussed. From the point of view of the first evaluator: (A) the subject of the study is fascinating and practical. (B) The exercise is excellent and it can be performed in natural conditions. From the point of view of the second evaluator: (A) Time management, the use of two expert nurses experienced in the Bam earthquake, the use of the emergency team with work experience of 2–25 years and their good cooperation, sound quality and the use of clinical skill space for the exercise were the strengths of the exercise. (B) The weaknesses of the exercise included audio–video disconnection, the insufficient mastery of the nurses in working with Skype software and the lack of facilities provided for experts.

## Discussion

The results of the study showed that the mean score of possibility of telenursing in disasters was at a high level, which confirmed the provision of telenursing services in disasters. The results of the drill evaluation showed that in the simulated earthquake scenario in this study, inexperienced nurses could implement and operationalize nursing care for the injured by using information and communication tools. This result consist with the study findings of Heo et al. [13] Who stated that health system management should establish a telenursing system capable of managing citizens' health conditions in everyday situations and then adjusting appropriately in disasters. To achieve this goal, protocols and guidelines for the organization and operation of nursing resources must be developed.

According to the result of drill evaluation, mutual communication and interactions were well established and the nurses participating in the executive phase of the study could use the technology provided on the internet to receive and implement the instructions provided by experts in caring for hypothetical trauma victims. This result consist with the findings of a study conducted by Xiong et al. [19] who assessed the impact of using telemedicine to provide emergency specialty care to patients at local hospitals on medical surge capacities at both the local and regional levels and declared a regional telemedicine hub, which includes linkage of a telemedicine command center with an extended network of clinical experts in the event of a natural or intentional disaster, which may facilitate future disaster response and improve patient outcomes. Telenursing has strengthened nursing activities by incorporating a communication system into areas such as education, research, and management [20]. Telenursing has most commonly used telephone and video calls, websites, and other forms of information technology to collect patient information and transmit data to healthcare workers [21].

Our drill evaluation showed the telenursing could improve the delivery of health care by increasing access to services bringing specialist expertise to the victims. This results consist with study of Heo et al. [13] That conducted in South Korea, to develop the first telenursing service for covid-19 patients and their declared that policies for appropriate and prompt healthcare interventions in emergencies and disasters through telenursing should be developed to respond to the rapidly changing healthcare environment. Ajami and Lamoochi in a study reported that using of telemedicine systems such as telenursing, telemonitoring, tele-radiology and videoconferencing could be promotion prehospital and hospitals response to emergencies and disasters. In addition, their declared that the telemedicine can encourage appropriate measures to respond to three major events, including before the incident, incident, and rehabilitation [22]. The result of studies [9, 16, 23, 24] showed that telenursing in disasters was the turning point of the care management of victims and the nursing paradigm in the twenty-first century global era has been developed with the help of technology to meet the needs of distance efficiency and cost limitations. Thus, telenursing is a solution to answer these challenges.

In a study titled "Challenges and Opportunities for Telehealth During the COVID-19 Pandemic: Ideas on Spaces and Initiatives in the Brazilian Context," Rosângela Caetano et al. reported that telehealth provides capabilities for remote screening, care, and treatment, as well as monitoring, surveillance, detection, prevention, and mitigation of the impacts on healthcare indirectly related to COVID-19. The initiatives sparked by this process have the potential to reshape the future space of telemedicine in health services in the territory [25].

## Limitation

The present study had some limitations. First, implementing the exercise in the form of simulation required comprehensive coordination with managers and health professionals in disasters and emergencies, as well as the need to allocate time and enough financial resources. Second, this study was conducted in a small place with using the micro hypothetical incident scenario for the first time in Iran in the form of the pilot project, therefore our study suggests that in the future the possibility of nursing interventions through telemedicine in disaster risk management performing in the high-level incidents through performing of discussion-based and operation-based exercise.

## Conclusion

Today, disaster management is almost impossible without the use of new technologies. This study found that due to the lack of specialized nursing staff in the deprived areas affected by disasters, the most crucial way to provide health care for a large group of the population is to develop effective health services so that everyone can use these services equally and fairly. Therefore, the establishment and expansion of the telemedicine system to develop health services in these areas can be a good solution to solve this problem. Nurses should be trained how to use new communication and information technologies during their studies to provide better services at the scene. Based on the results of this study, we propose that medical informatics content should be included in the nursing education curriculum and performing research studies on the development of guidelines for telenursing care in disaster risk management.

## Data Availability

The datasets generated and/or analyzed during the current study are not publicly available due to restrictions of the Ethics Committee of Kerman University of Medical Sciences. For available data, please contact: kmu_Research@yahoo.com.
